# Supplementary material for: GV1001 Inhibits the Severity of the Ligature-Induced Periodontitis and the Vascular Lipid Deposition Associated with the Periodontitis in Mice
Source: Int J Mol Sci. 2023 Aug 8;24(16):12566. doi: 10.3390/ijms241612566 (PMC10454325; doi:10.3390/ijms241612566)
Supplement: Supplementary file 1 [file ijms-24-12566-s001.zip › ijms-2525903-supplementary.pdf]

Supplemental Materials

Article

GV1001 Inhibits the Severity of the Ligature-Induced Periodontitis and the Vascular Lipid Deposition Associated with the Periodontitis in Mice

Sharon Y Kim [1,†], Yun-Jeong Kim [2,†], Suyang Kim [1], Mersedeh Momeni [1], Alicia Lee [1], Alexandra Treanor [1,‡], Sangjae Kim [3], Reuben H Kim [1,4], and No-Hee Park [1,4,5,*]

[1] The Shapiro Family Laboratory of Viral Oncology and Aging Research, UCLA School of Dentistry, 714 Tiverton Ave, Los Angeles, CA 90095, USA
[2] Department of Periodontology, Seoul National University Gwanak Dental Hospital, School of Dentistry and Dental Research Institute, Seoul National University, Seoul 08826, Republic of Korea
[3] Teloid Inc., 920 Westholme Avenue, Los Angeles, CA 90024, USA
[4] UCLA Jonsson Comprehensive Cancer Center, 10833 Le Conte Ave, Los Angeles, CA 90095, USA
[5] Department of Medicine, David Geffen School of Medicine at UCLA, 10833 Le Conte Ave, Los Angeles, CA 90095, USA
* Author to whom correspondence should be addressed.
† These authors contributed equally to this study
‡ Present Address: Marist School, 3790 Ashford Dunwoody Rd, NE, Atlanta, GA 30319, USA

Running title: GV1001 inhibits the severity of periodontitis and atherosclerosis

Correspondence:
No-Hee Park, DMD, PhD
43-005 CHS, Box 951668, University of California, Los Angeles, CA 90095-1668
310-825-0339 (voice), nhpark@g.ucla.edu (email)

Keywords: GV1001; periodontitis; systemic and vascular inflammation; atherosclerosis

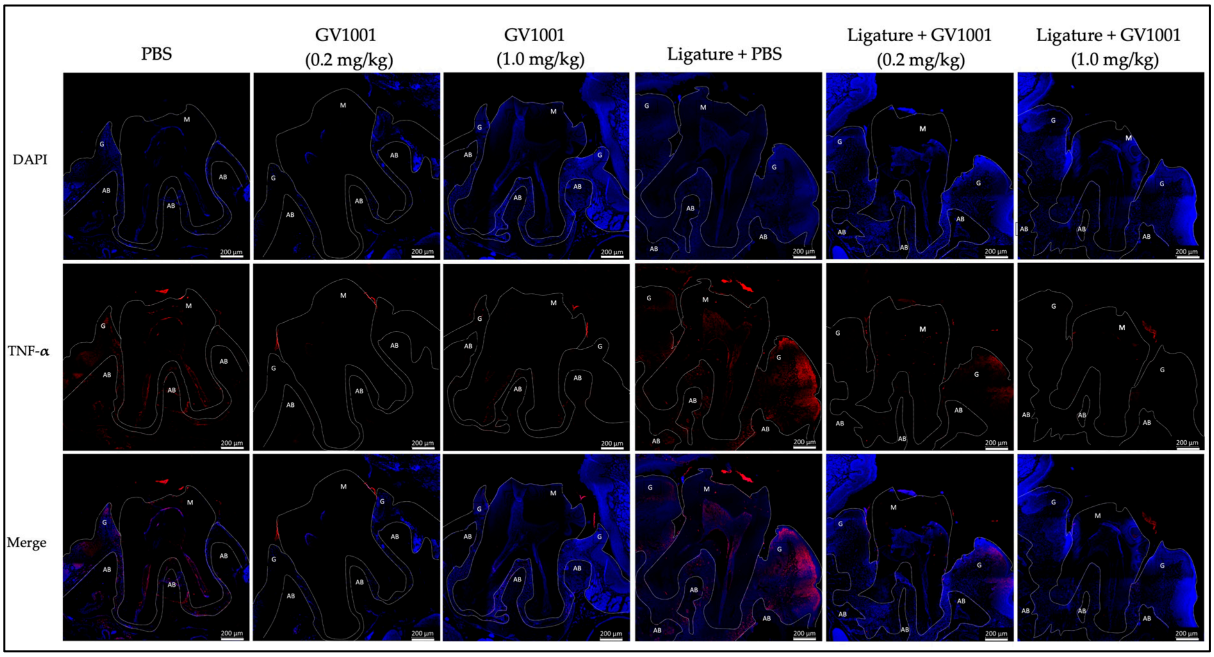

Figure S1. Representative immunofluorescent staining of p50 subunit, an NF*κ*B/Rel family, of gingival tissue. A low level of p50 was found in the gingival tissue of control mice receiving PBS only, and the protein level was reduced by GV1001 administration. Ligature placement enormously enhanced the level of p50 in the tissue, and the GV1001 administration remarkably inhibited the increase. Notably, a high dose of GV1001 (1.0 mg/kg) completely negated the increase induced by the ligature placement. The p50 was primarily located in the cytoplasm of cells in the control and groups receiving GV1001 (see Merge). AB: Alveolar Bone; G: Gingival tissue; M: Molar.

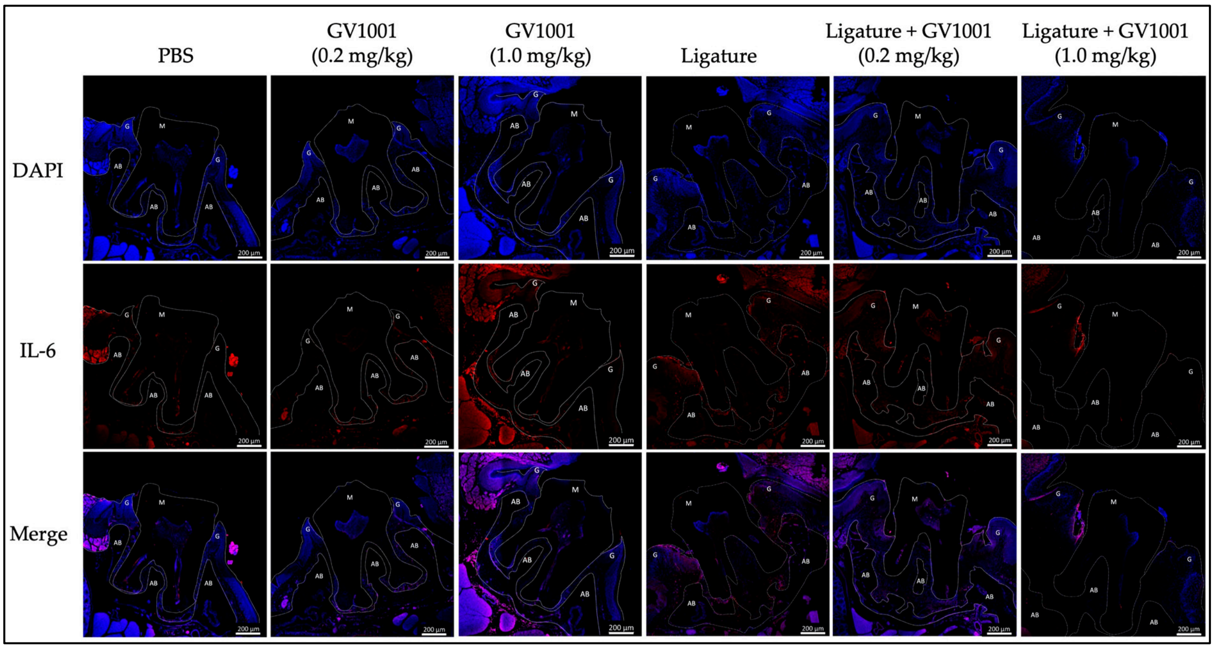

Figure S2. Representative immunofluorescent staining of IL-6 of gingival tissue. A very low level of IL-6 was found in the gingival tissue of control mice receiving PBS only, and the protein level was not altered by GV1001 administration. Ligature placement enhanced the level of IL-6 in the tissue, and the GV1001 administration, particularly the higher dose, notably reduced the increase induced by the ligature placement.

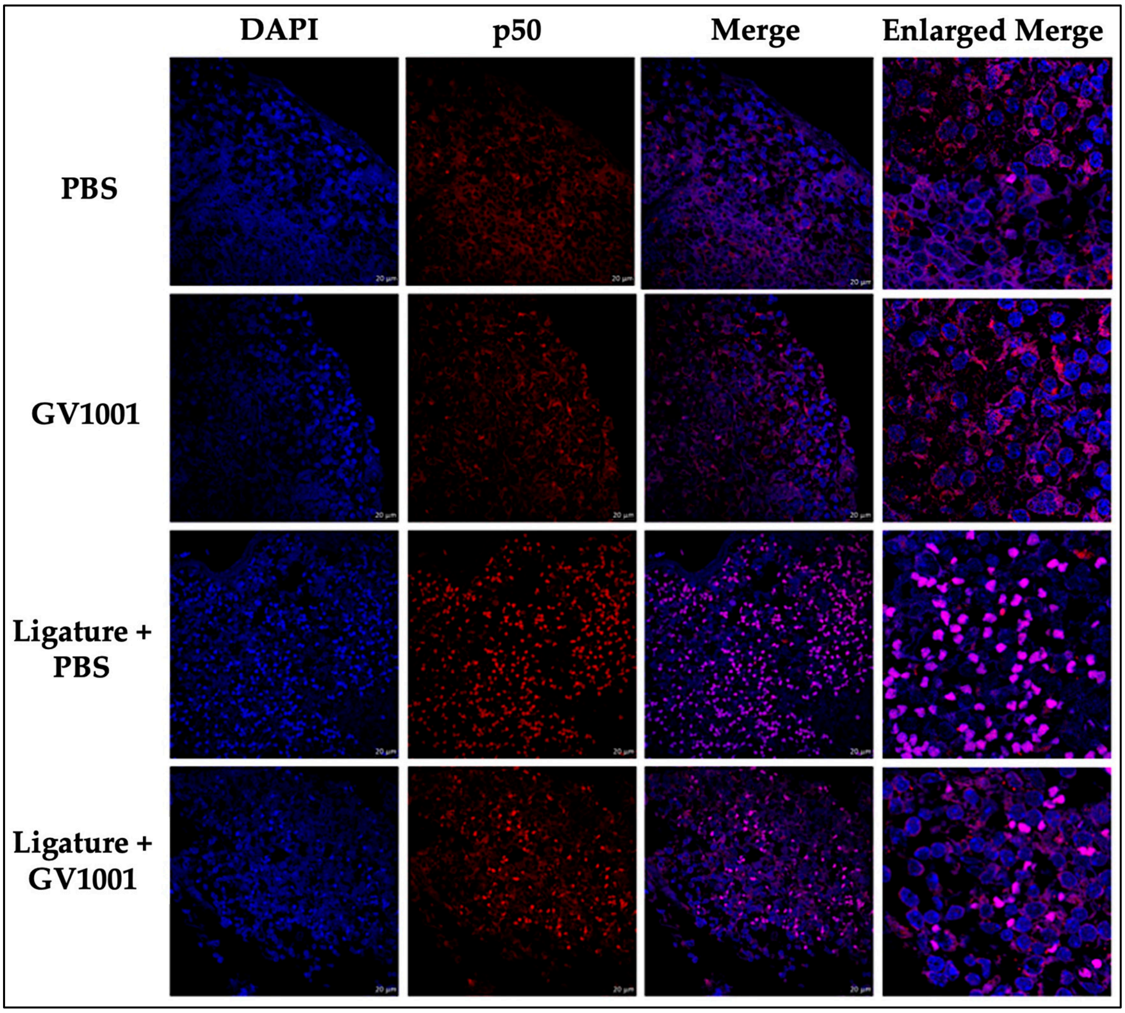

Figure S3. Representative immunofluorescent staining of NFκB p50 in the spleen. The spleen cells of mice receiving ligature placement showed that p50 was translocated into the nucleus, which is reversed by GV1001 (1.0 mg/kg) administration. Magnifications of the pictures were ×200, and those of the merged pictures were ×600.

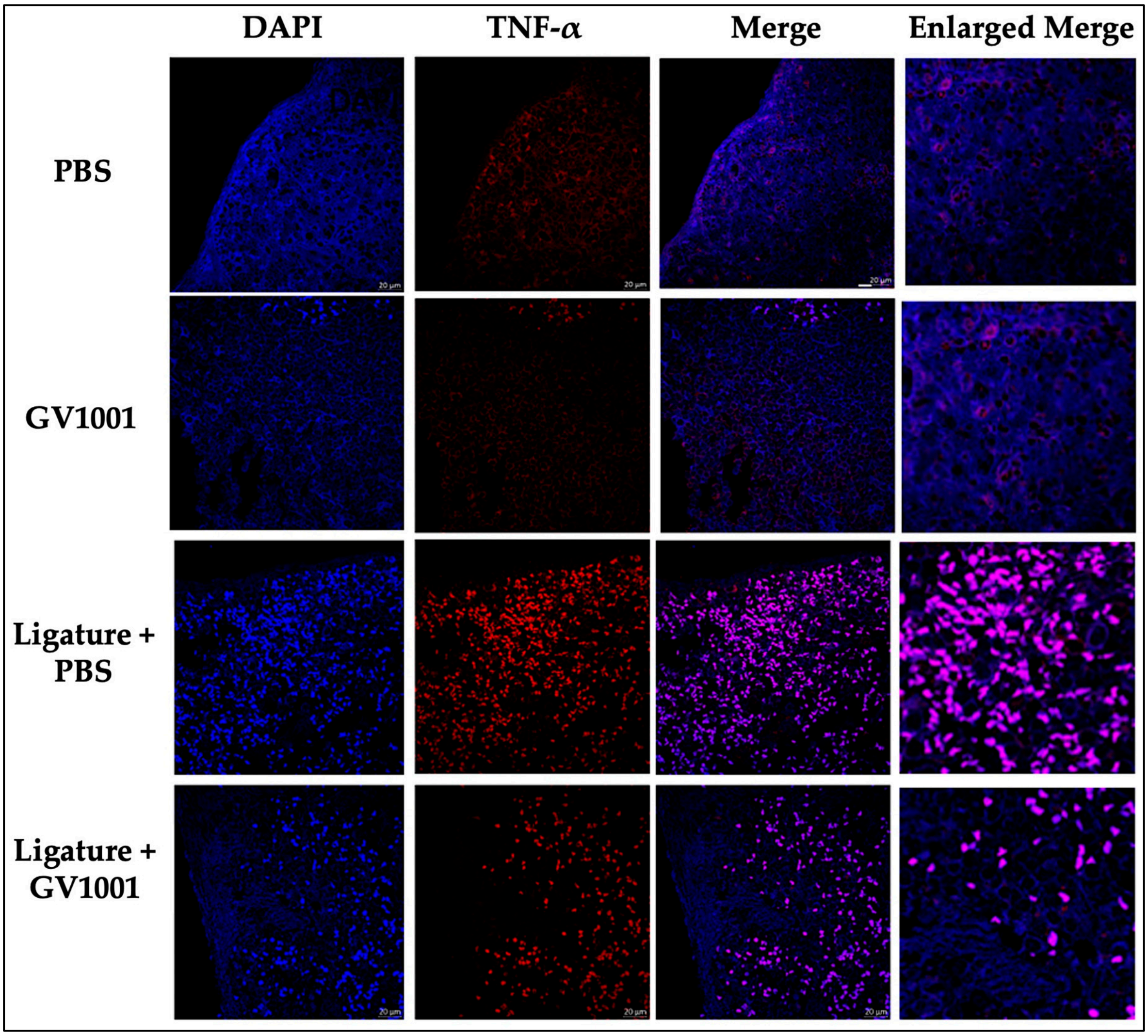

Figure S4. Representative immunofluorescent staining of TNF-α in the spleen. The spleen cells of mice receiving ligature placement showed a high level of TNF-α, which is reversed by GV1001 (1.0 mg/kg) administration. Magnification of the pictures were ×200 and those of the enlarged merge were ×600.

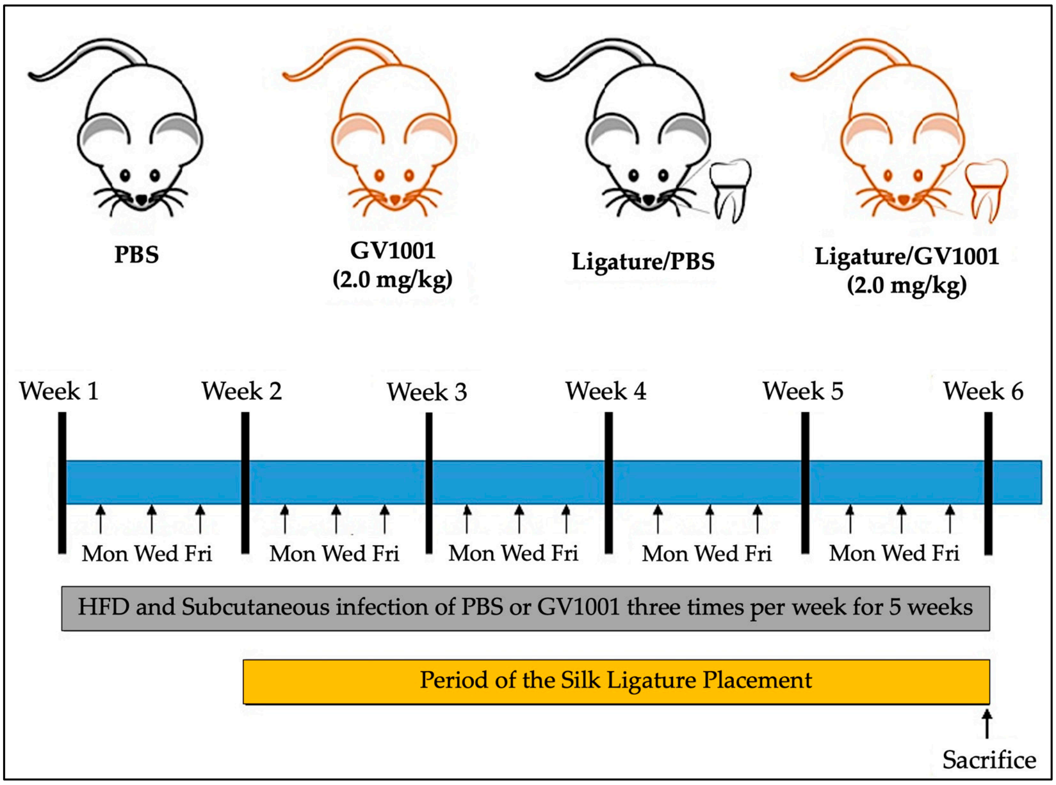

Figure S5. Experimental design for the effect of GV1001 on the exacerbated development atherosclerosis by the ligature placement in *ApoE*-deficient mice. Nine-week old mice were divided into four groups: (1) Control: PBS injection three times per week for 5 weeks; (2) Mice receiving GV1001 (2.0mg/kg) injection 3 times per week for 5 weeks ; (3) Mice receiving PBS injection 3 times per week for 5 weeks with the ligature placement on 1 week after the initiation of PBS injection; (4) Mice receiving GV1001 (2.0mg/kg) s.c. injection 3 times per week for 5 weeks and silk ligature placement 1 week after the initiation of GV1001 injection.

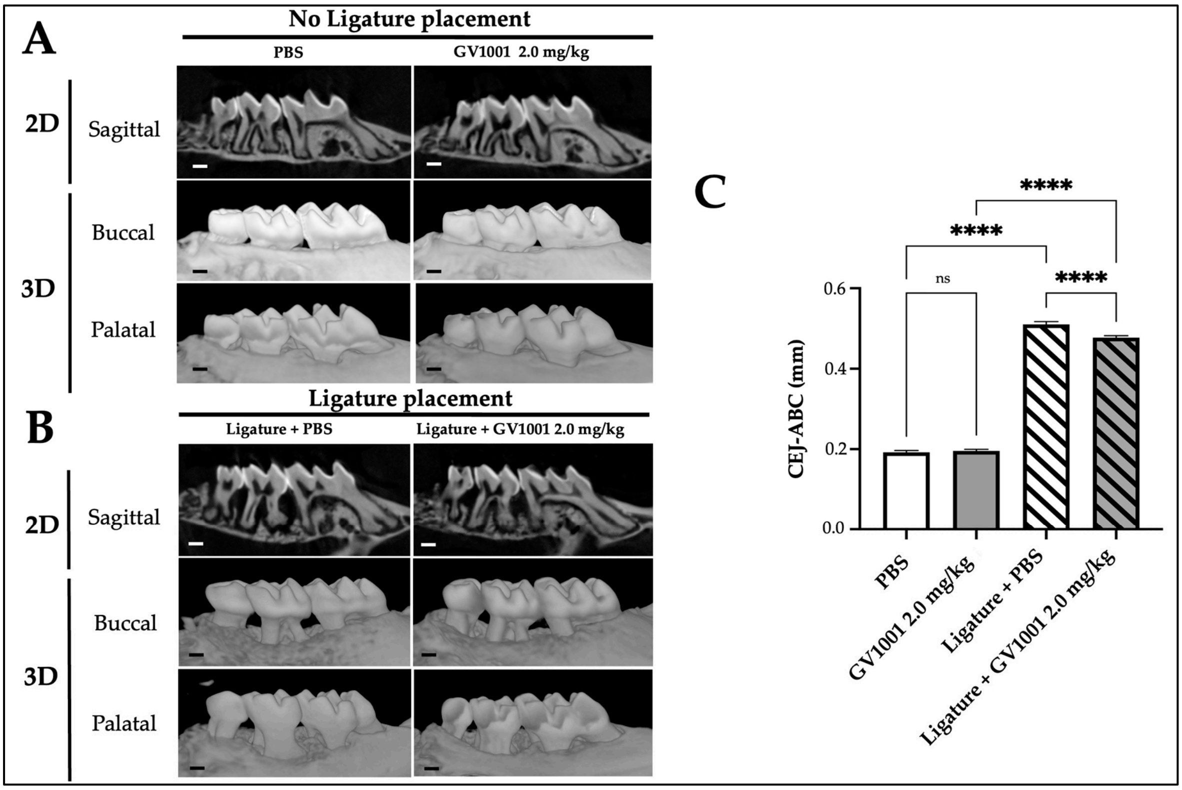

Figure S6. Representative two or three dimensional μCT images of mice maxillae in *ApoE*-deficient mice. **A.** Mice with no ligature placement and with/without GV1001 injection. **B.** mice with ligature placement and with/without GV1001 injection. Scale bar: 0.2 mm. **C.** The average distance (unit; mm) from the palatal and buccal CEJ to the ABC of second molar. Results represent the means ± SEM performed in ten samples. **** $p < 0.0001$. ns—not significantly differernt between two groups ($p > 0.05$); CEJ, cemento-enamel junction; ABC, alveolar bone crest.

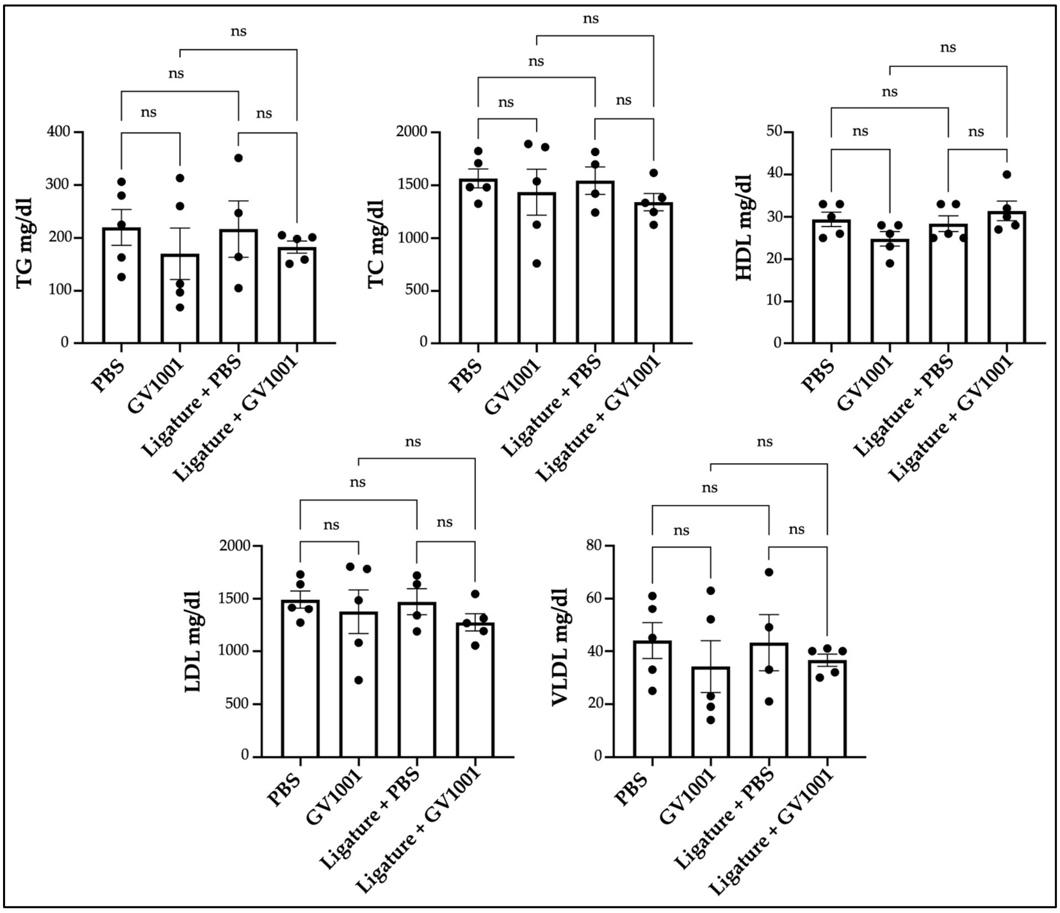

Figure S7. The ligature placement and GV1001 did not alter serum cholesterol profiles in *ApoE*-deficient mice **TG:** Levels of Serum lipid; triglyceride, **TC**: total cholesterol, **HDL**: high-density lipoprotein, **LDL**: low-density lipoprotein, and **VLDL**: very low-density lipoprotein. ns - not significantly different between two groups.

Supplemental Table S1. Primer sequences of IL-6, -IL-1b, TNF-α, iNOS, and GAPDH.

**Primers**	**Sequences**
Il-6-F	TGGGACTGATGCTGGTGACA
Il-6-R	GCCTCCGACTTGTGAAGTGGT
IL-1β-F	CACAGCAGCACATCAACAAG
IL-1β-R	GTGCTCATGTCCTCATCCTG
TNFα-F	TCAGGTTGCCTCTGTCTCAG
TNFα-R	GCTCTGTGAGGAAGGCTGTG
GAPDH-F	AGCTTGTCATCAACGGGAAG
GAPDH-R	TTTGATGTTAGTGGGGTCTCG
